# A Cross-Sectional Study on Corrected-Term Screening for Osteopenia/Metabolic Bone Disease of Prematurity in Preterm and Low-Birth-Weight Neonates: Prevalence, Risk Factors, and Feeding-Related Predictors

**DOI:** 10.7759/cureus.111595

**Published:** 2026-06-27

**Authors:** Amtul Saleem Asfa, Kagithapu Surender, Chitra Lekha B S, T Jaya Chandra

**Affiliations:** 1 Pediatrics, Modern Government Maternity Hospital, Petlaburj, Hyderabad, IND; 2 Pediatrics, Government Medical College, Jayashankar Bhupalpally, Jayashankar Bhupalpally, IND; 3 Pediatrics, Government Medical College, Vikarabad, Vikarabad, IND; 4 Microbiology, GSL Medical College, Rajahmundry, IND

**Keywords:** alkaline phosphatase, enteral feeding, metabolic bone disease, osteopenia of prematurity, preterm neonate

## Abstract

Background: Osteopenia/metabolic bone disease of prematurity (MBDP) is an important morbidity among preterm and very-low-birth-weight neonates due to reduced third-trimester mineral accretion and postnatal nutritional challenges.

Aim: This study aimed to find the prevalence of osteopenia/MBDP in preterm neonates born before 34 weeks of gestation and/or with birth weight <1500 g.

Methods: This cross-sectional observational research was conducted in the Department of Pediatrics of Mahatma Gandhi Memorial Hospital in Warangal, India, from September 2022 to April 2024. A total of 110 eligible preterm neonates admitted to the neonatal intensive care unit (NICU) were included. Demographic variables, neonatal risk factors, respiratory support, total parenteral nutrition (TPN) exposure, caffeine use, feeding details, biochemical parameters, and radiological findings were recorded. Data analysis was carried out using IBM SPSS Statistics for Windows, Version 25.0 (IBM Corp., Armonk, New York, United States).

Results: Osteopenia/MBDP was diagnosed in 22 neonates, giving a prevalence of 20%. Lower birth weight, lower gestational age, small for gestational age (SGA) status, continuous positive airway pressure (CPAP) >5 days, mechanical ventilation >5 days, TPN >1 week, caffeine citrate >20 days, and delayed enteral feeding were associated with disease.

Conclusion: Osteopenia/MBDP is common among preterm neonates and is strongly associated with delayed feeding and neonatal illness severity.

## Introduction

Osteopenia/metabolic bone disease of prematurity (MBDP) is an important morbidity among very preterm and very-low-birth-weight (BW) neonates. The condition is often clinically silent in early life, while biochemical abnormalities such as hypophosphatemia and raised alkaline phosphatase (ALP) may precede obvious radiological osteopenia or fractures. Recent Indian evidence has shown that metabolic bone disease (MBD) is frequent among preterm infants, with lower BW and feeding-related factors being important predictors [1]. Another Indian observational study reported that delayed attainment of full enteral feeding was associated with MBD, supporting the need to evaluate feeding practices along with biochemical markers [2]. Screening protocols using serum calcium, phosphorus, and ALP have also been recommended for infants born <1500 g or <32 weeks of gestation because early detection may allow timely nutritional intervention [3]. Nutritional studies further support the importance of calcium, phosphorus, and feeding-related factors in the development or prevention of osteopenia/MBDP [4,5]. Literature from the newly formed Telangana state on MBDP is limited. Therefore, the present research aims to determine the osteopenia/MBDP prevalence among preterm neonates born <34 weeks of gestation and/or with BW <1500 g at a corrected gestational age (GA) of 40 weeks. The primary objective of this study was to determine the prevalence of osteopenia/MBDP among preterm neonates born before 34 weeks of gestation and/or with BW <1500 g, while the secondary objectives were to identify neonatal and treatment-related risk factors associated with the disease, evaluate the association between enteral feeding practices and osteopenia/MBDP, and assess the relationship between biochemical markers and radiological Knee Injury and Osteoarthritis Outcome Score (KOOS) findings.

## Materials and methods

This cross-sectional observational study was conducted in the Department of Pediatrics of Mahatma Gandhi Memorial Hospital in Warangal, India, from September 2022 to April 2024. The study population included preterm neonates of both sexes admitted to the neonatal intensive care unit (NICU) during the study period. All preterm infants with GA <34 weeks and/or BW <1500 g were considered eligible for enrolment. Purposive sampling of all consecutive eligible neonates was followed. Neonates were excluded if they died, were referred to another center within the first six weeks of life, or had major congenital anomalies, surgical conditions, liver impairment, renal impairment, suspected primary bone disease, or muscular disorders. Preterm birth was defined as birth before the completion of 37 weeks of gestation. Based on GA, neonates were classified as preterm, term, and post-term; based on BW, they were classified as extremely low BW, very low BW, and low BW [6]. Since this was a time-bound hospital-based observational study, all eligible neonates admitted during the study period were included.

Data collection was initiated after obtaining approval from the Kakatiya Institutional Ethics Committee (approval number: KIEC/PG DISS-2021-22/32). Written informed consent was obtained from parents or legal guardians before enrolment. Eligible neonates were evaluated using a predesigned and pretested proforma. Details regarding sex, GA, BW, antenatal risk factors, neonatal clinical course, respiratory support, duration of continuous positive airway pressure (CPAP) therapy, mechanical ventilation, total parenteral nutrition (TPN) exposure, caffeine citrate therapy, feeding details, time of initiation of enteral feeding, time to establishment of full enteral feeds, and duration of hospital stay were recorded. Feeding-related variables included timing of initiation of enteral feeds, delayed initiation of feeds, use of formula feeds, and milk fortification wherever applicable. Screening for osteopenia/MBDP was performed around six weeks of postnatal age/corrected-term assessment, depending on clinical stability and follow-up availability.

Osteopenia/MBDP was defined using biochemical and radiological parameters. Biochemical evaluation included serum calcium, serum phosphorus, and serum ALP. Raised serum ALP >700 IU/L and low serum phosphorus were considered suggestive of MBDP. Radiological assessment was performed using X-ray findings graded by the KOOS scoring system. Neonates with biochemical abnormality supported by radiological evidence of reduced bone mineralization were classified as having osteopenia/MBDP. Serum calcium and phosphorus values were also analyzed in relation to the KOOS radiological score. Although prolonged TPN was evaluated as a risk factor, the exact calcium and phosphorus composition of TPN was not consistently available in the records; therefore, TPN exposure was analyzed based on duration.

Data were analyzed using IBM SPSS Statistics for Windows, Version 25.0 (IBM Corp., Armonk, New York, United States). Categorical variables were expressed as frequencies and percentages, while continuous variables were expressed as mean±standard deviation (SD). The prevalence of osteopenia/MBDP was calculated as the proportion of affected neonates among the total study population. Associations between categorical risk factors and osteopenia/MBDP were assessed using the chi-squared test. Fisher's exact test was applied for 2×2 tables when the expected cell counts were small. For variables with more than two categories and sparse cell frequencies, results were interpreted cautiously. Unadjusted odds ratios (OR) with 95% confidence intervals (CI) were calculated for selected binary predictors. Multivariable logistic regression was not performed because of the limited number of outcome events and sparse cell counts, which could have resulted in unstable adjusted estimates. A p-value of <0.05 was considered statistically significant.

## Results

Study population and prevalence of MBDP

A total of 110 preterm neonates were included in the study. Osteopenia/MBDP was diagnosed in 22 neonates, giving an overall prevalence of 20%. The association of neonatal characteristics, treatment-related factors, and feeding-related variables with osteopenia/MBDP was analyzed and is summarized in Tables 1-3.

**Table 1:** Association of neonatal characteristics with osteopenia/MBD MBD: metabolic bone disease; GA: gestational age; AGA: appropriate for gestational age; SGA: small for gestational age

Variable	Category	Osteopenia/MBD, n (%)		Chi-square	P-value
		Present	Absent		
Birth weight	<1 kg	2 (50)	2 (50)	11.302	0.01
	1-1.5 kg	11 (34.4)	21 (65.6)		
	1.6-2 kg	8 (17.4)	38 (82.6)		
	>2 kg	1 (3.6)	27 (96.4)		
GA	28-30 weeks	11 (28.9)	27 (71.1)	12.284	0.007
	31-32 weeks	7 (17.9)	32 (82.1)		
	33-34 weeks	4 (12.1)	29 (87.9)		
Growth status	AGA	17 (18.8)	73 (81.1)	10.99	0.03
	SGA	5 (25)	15 (75)		
Gender	Male	9 (18)	41 (82)	3.284	0.194
	Female	13 (21.6)	47 (78.3)		

**Table 2:** Association of treatment and feeding with osteopenia/MBD MBD: metabolic bone disease; CPAP: continuous positive airway pressure; TPN: total parenteral nutrition

Variable	Category/comparison	Osteopenia/MBD, n (%)		Test value	P-value
		Present	Absent		
CPAP >5 days	Yes	16 (29.6)	38 (70.4)	χ²=6.142	0.013
	No	6 (10.7)	50 (89.3)		
Mechanical ventilation >5 days	Yes	8 (26.7)	22 (73.3)	χ²=5.022	0.025
	No	14 (17.5)	66 (82.5)		
TPN >1 week	Yes	14 (28)	36 (72)	χ²=8.250	0.004
	No	8 (13.3)	52 (86.7)		
Start of enteral feeds	<3 days	0 (0)	31 (100)	χ²=51.200	<0.001
	3-7 days	10 (15.2)	56 (84.8)		
	>7 days	12 (92.3)	1 (7.7)		
Caffeine citrate duration	7-14 days	6 (13)	40 (87)	χ²=6.764	0.040
	15-20 days	6 (13.6)	38 (86.4)		
	>20 days	10 (50)	10 (50)		

**Table 3:** Unadjusted odds ratios for factors associated with osteopenia/MBDP MBDP: metabolic bone disease of prematurity; BW: birth weight; GA: gestational age; AGA: appropriate for gestational age; GA: small for gestational age; CPAP: continuous positive airway pressure; TPN: total parenteral nutrition; FET: Fisher's exact test

Risk factor	Comparison	Unadjusted OR	95% CI	FET p-value
BW	<1.5 vs. ≥1.5 kg	4.08	1.54-10.81	0.005
GA	28-30 vs. 31-34 weeks	2.26	0.87-5.85	0.131
SGA status	SGA vs. AGA	1.43	0.46-4.48	0.544
Gender	Female vs. male	1.26	0.49-3.25	0.811
CPAP duration	>5 vs. ≤5 days	3.51	1.25-9.82	0.017
Mechanical ventilation	>5 vs. ≤5 days	1.71	0.63-4.63	0.295
TPN duration	>1 vs. ≤1 week	2.53	0.96-6.65	0.092
Caffeine citrate	>20 vs. ≤20 days	6.5	2.24-18.89	0.001
Enteral feeding	>7 vs. ≤7 days	104.4	12.25-889.47	<0.001

Baseline characteristics

Most neonates were evaluated between 28 and 34 days of postnatal age, accounting for 75 cases (68.2%). The mean postnatal age was 33.84±8.35 days. The mean GA was 31.45±1.73 weeks, and the mean BW was 1.75±0.38 kg. Female neonates constituted 54.5% of the study population, and 18.2% were small for gestational age (SGA) (Table 1).

Association of neonatal characteristics with MBDP

Prolonged respiratory and nutritional support were significantly associated with osteopenia/MBDP. Neonates who required CPAP for >5 days showed a significant association with MBDP (p=0.013). Similarly, mechanical ventilation for >5 days (p=0.025), TPN for >1 week (p=0.004), and caffeine citrate therapy for >20 days (p=0.040) were significantly associated with the development of MBDP (Table 2). In addition, the overall mean serum calcium and phosphorus levels were 8.210±0.71 mg/dL and 4.05±1.11 mg/dL, respectively; however, the exact calcium and phosphorus composition of TPN was not consistently available in the records, and, therefore, TPN exposure was analyzed based on duration rather than mineral content.

Association of treatment-related factors with MBDP

Prolonged respiratory and nutritional support were significantly associated with osteopenia/MBDP. Neonates who required CPAP for >5 days showed a significant association with MBDP (p=0.013). Similarly, mechanical ventilation for >5 days (p=0.025), TPN for >1 week (p=0.004), and caffeine citrate therapy for >20 days (p=0.040) were significantly associated with the development of MBDP (Table 2).

Association of enteral feeding with MBDP

Delayed initiation of enteral feeding showed the strongest association with osteopenia/MBDP. Among neonates in whom enteral feeds were started after seven days, 12 neonates (92.3%) developed MBDP. This association was statistically highly significant (p<0.001; Table 2), suggesting that delayed enteral nutrition may be an important feeding-related predictor of MBD in preterm neonates.

Unadjusted risk estimates

Table 3 presents the unadjusted risk estimates for factors associated with MBDP. BW <1.5 kg, CPAP support for >5 days, caffeine citrate therapy for >20 days, and delayed initiation of enteral feeding beyond seven days showed significant associations with osteopenia/MBDP. Among these, delayed enteral feeding had the strongest association, with markedly higher odds of disease.

## Discussion

In this research, the prevalence of osteopenia/MBDP was 20% (22/110). It was clinically relevant because MBDP is often silent in the early neonatal period and may first be suspected through biochemical or radiological abnormalities rather than obvious skeletal signs. Recent Indian data also support that MBDP remains a significant morbidity in preterm infants. Krishna et al. reported moderate MBDP in 14.4% and severe MBDP in 18.3% of Indian preterm infants born at ≤33 weeks, showing that nearly one-third of such infants may develop bone disease [1]. Similarly, Kolisambeevi et al. found that implementation of bone-protective nutritional strategies could reduce the MBD burden among infants born before 30 weeks, highlighting the preventable component of the disease [7]. The available data from Telangana on this remains limited; hence, the present study adds useful regional data from a tertiary care special newborn care unit (SNCU)/NICU setting [8].

Lower BW showed a statistically significant association in this study, with the highest (50%) prevalence among neonates weighing <1 kg (Table 1). This pattern is biologically plausible because extremely-low-BW and very-low-BW neonates have reduced fetal mineral accretion, limited postnatal nutrient reserves, prolonged intensive care exposure, and delayed advancement of mineral-rich feeds. The Indian prospective study by Krishna et al. also found lower BW to be associated independently with severe MBDP [1]. International evidence also supports this; Wang et al., in a meta-analysis, identified BW <1000 g and GA <32 weeks as important risk factors for MBDP [9]. Avila-Alvarez et al. found that infants who developed MBD had lower GA and BW and longer exposure to parenteral nutrition and hospitalization [10]. In this study, GA was also significantly associated with MBD, with the highest prevalence among neonates born at 28-30 weeks (28.9%; Table 1). This finding is consistent with the known physiology of fetal mineralization, because the majority of calcium and phosphorus transfer occurs during the third trimester; therefore, early delivery interrupts the period of maximal skeletal mineral deposition [5]. These observations support the need for special surveillance in neonates with very low GA and BW [5,9,10].

We also observed a significant correlation between SGA and osteopenia/MBD, whereas gender was not significantly associated (Table 1). SGA neonates are vulnerable because placental insufficiency, intrauterine growth restriction, and reduced fetal nutrient transfer may impair mineral stores before birth. Although SGA status is not always uniformly reported as an independent predictor, it is biologically compatible with impaired bone mineralization, especially when combined with prematurity and poor postnatal growth. As per Wang et al., intrauterine growth restriction was associated with MBDP, supporting the relevance of growth restriction in the risk profile [9]. In the current study, female infants constituted 54.5%, but the difference in MBD prevalence between genders was statistically not significant (χ²=3.284; p=0.194; Table 1). As per this finding, gender alone may not be a major determinant of osteopenia of prematurity when compared with stronger clinical determinants such as GA, BW, nutrition, sepsis, respiratory morbidity, and parenteral nutrition. Similar conclusions were noted in previous observational studies where neonatal illness severity and nutritional variables showed stronger associations than gender-based differences [10,11]. Therefore, screening strategies should not be restricted by gender, and all eligible preterm or very-low-BW infants should be evaluated [2,11].

Respiratory morbidity and prolonged intensive care support were important risk factors in this study. Neonates requiring CPAP for >5 days (16; 29.6%) and mechanical ventilation for >5 days (8; 26.7%) had significantly higher prevalence of MBD (Table 2). This association may reflect the combined effect of lower GA, illness severity, immobilization, delayed feeding, prolonged hospitalization, and exposure to drugs such as caffeine, steroids, and diuretics. MBDP is multifactorial, and critically ill preterm infants often accumulate multiple risk exposures. Chacham et al. emphasized that adequate calcium, phosphorus, vitamin D supplementation, optimum nutrition, and physiological activity are important components in the prevention and management of MBD in premature neonates [12]. Rehman and Narchi also noted that high-risk infants include preterm infants, those receiving corticosteroids or diuretics, and those with neuromuscular or chronic illness-related immobility [13]. The present finding that caffeine citrate use for >20 days was associated with MBD (10; 50%) may not indicate a direct role alone, but may act as a marker of prolonged apnea, respiratory immaturity, and overall neonatal illness burden (Table 2). Therefore, neonates requiring prolonged respiratory support should be treated as a high-risk group for nutrition optimization [12,13].

Nutritional factors showed the strongest association with osteopenia/MBD in this study. TPN for >1 week was significantly associated with MBD, and delayed initiation of enteral feeds showed the strongest relationship; 92.3% (12) of neonates whose enteral feed started >7 days developed osteopenia/MBD (Table 2). This finding is directly relevant to the study objective on enteral feeding and is supported by recent Indian evidence. Kar et al. reported that delayed attainment of full enteral feeding beyond seven days was a significant predictor of MBD in very preterm neonates [2]. Pillai et al. also reported delayed attainment of full feeds and delayed mineral supplementation as independent risk factors for MBD in preterm infants [14]. Kolisambeevi et al. showed that early phosphorus use, early fortification, and weekly phosphorus monitoring as part of a bone-protective nutrition strategy could reduce MBD burden in preterm neonates [7]. These suggest that early enteral feeding, timely human milk fortification, and calcium-phosphorus supplementation are central preventive strategies. The current study results, therefore, suggest that feeding delay is not only a nutritional variable but also a clinically measurable predictor of MBD risk in the NICU setting [2,7,14].

The study has several important strengths, including its clinical relevance, clearly defined target population, and evaluation of multiple neonatal, treatment-related, and feeding-related risk factors for osteopenia/MBDP. However, certain methodological limitations should be considered while interpreting the findings. The cross-sectional design limits the ability to establish causal relationships, and the absence of adjusted multivariable analysis restricts the assessment of independent predictors. In addition, incomplete reporting of some diagnostic and nutritional variables, including detailed calcium and phosphorus content of TPN, may have influenced interpretation.

## Conclusions

Osteopenia/MBDP was identified in 20% of preterm neonates in this cohort. Lower GA, lower BW, SGA status, prolonged CPAP, mechanical ventilation, TPN for more than one week, prolonged caffeine citrate therapy, and delayed initiation of enteral feeding were associated with MBDP. Delayed enteral feeding beyond seven days showed the strongest observed unadjusted association with MBDP in this study; however, this finding should be interpreted cautiously, as it may be influenced by prematurity, illness severity, respiratory support, and prolonged TPN. These findings suggest the need for early nutritional assessment, timely advancement of enteral feeding where clinically feasible, and risk-based biochemical and radiological screening in preterm neonates. The study was limited by its single-center cross-sectional design, purposive sampling, absence of adjusted multivariable analysis, lack of long-term follow-up, and incomplete availability of detailed nutritional variables such as calcium and phosphorus content of TPN.
